# Style attention based global-local aware GAN for personalized facial caricature generation

**DOI:** 10.3389/fnins.2023.1136416

**Published:** 2023-03-07

**Authors:** Xiuzhi Zhao, Wenting Chen, Weicheng Xie, Linlin Shen

**Affiliations:** ^1^College of Artificial Intelligence, Zhejiang Industry & Trade Vocational College, Wenzhou, Zhejiang, China; ^2^Department of Electrical Engineering, City University of Hong Kong, Kowloon, Hong Kong SAR, China; ^3^Computer Vision Institute, School of Computer Science & Software Engineering, Shenzhen University, Shenzhen, China; ^4^Guangdong Key Laboratory of Intelligent Information Processing, Shenzhen University, Shenzhen, China

**Keywords:** caricature generation, individualized caricature generation, image generation, style transfer, shape exaggeration, GAN, image translation

## Abstract

**Introduction:**

Caricature is an exaggerated pictorial representation of a person, which is widely used in entertainment and political media. Recently, GAN-based methods achieved automatic caricature generation through transferring caricature style and performing shape exaggeration simultaneously. However, the caricature synthesized by these methods cannot perfectly reflect the characteristics of the subject, whose shape exaggeration are not reasonable and requires facial landmarks of caricature. In addition, the existing methods always produce the bad cases in caricature style due to the simpleness of their style transfer method.

**Methods:**

In this paper, we propose a Style Attention based Global-local Aware GAN to apply the characteristics of a subject to generate personalized caricature. To integrate the facial characteristics of a subject, we introduce a landmark-based warp controller for personalized shape exaggeration, which employs the facial landmarks as control points to warp image according to its facial features, without requirement of the facial landmarks of caricature. To fuse the facial feature with caricature style appropriately, we introduce a style-attention module, which adopts an attention mechanism, instead of the simple Adaptive Instance Normalization (AdaIN) for style transfer. To reduce the bad cases and increase the quality of generated caricatures, we propose a multi-scale discriminator to both globally and locally discriminate the synthesized and real caricature, which improves the whole structure and realistic details of the synthesized caricature.

**Results:**

Experimental results on two publicly available datasets, the WebCaricature and the CaVINet datasets, validate the effectiveness of our proposed method and suggest that our proposed method achieves better performance than the existing methods.

**Discussion:**

The caricatures generated by the proposed method can not only preserve the identity of input photo but also the characteristic shape exaggeration for each person, which are highly close to the real caricatures drawn by real artists. It indicates that our method can be adopted in the real application.

## 1. Introduction

**Figure 1:**
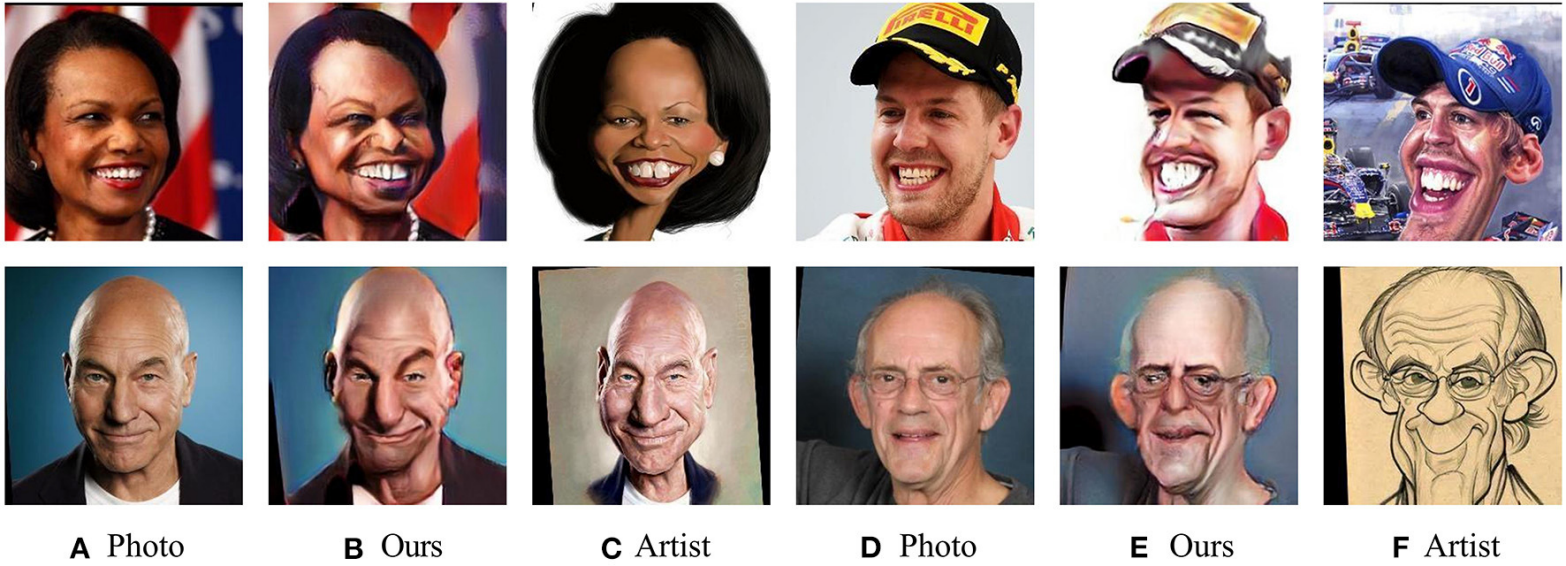
Example photos and caricatures of four subjects in our dataset. Columns **(A, D)** show each identity's real face photo, while four caricatures of the same subjects generated by our proposed methods are shown in columns **(B, E)**. Caricatures drawn by artists are shown in the columns **(C, F)**. We have obtained permission for the use of the images in this publication. Reproduced with permission from Nanjing University, available at https://cs.nju.edu.cn/rl/WebCaricature.htm.

Caricature represents a person or subject in an art form, which exaggerates individual characteristics to create a comic and grotesque effect (Redman, 1984). The characteristic of caricature can be humorous, comical, laughable, insulting or even offensive. Due to the characteristics of caricature, it has been widely used in different areas since a few decades ago. Newspapers and magazines always use caricatures of movie stars or politicians to criticize or praise them (Sadimon et al., 2010). In addition, caricature is also widely used in internet and mobile phone for social communication and entertainment. Figure 1 demonstrates some examples of photos and caricatures.

The features of caricature can be summarized into three main elements.

1. Likeness: Caricature should share the same identity as its subject, which needs to represent who it is.
2. Exaggeration: Caricature needs to exaggerate the facial features of its subject in a reasonable way, which highlights the unique characteristics of its subject.
3. Style: Caricature is a rendered image, which is non-photo realistic. It can preserve a variety of expressive styles like sketching, pencil strokes, oil painting, and other artistic styles.

In the past two decades, many researchers have been working on caricature generation. These methods can be divided into two groups, i.e., conventional methods and GAN-based methods. In conventional methods, most of them mainly focused on the style-specific caricature generation, such as facial sketch caricature (Chen et al., 2001; Liang et al., 2002; Mo et al., 2004), outline caricature (Fujiwara et al., 2000), and black-and-white illustration caricature (Gooch et al., 2004). In addition, some of conventional methods applied computer graphics techniques (Yang et al., 2016) and low-level geometric deformation (Akleman et al., 2000; Liu et al., 2006; Tseng et al., 2012) to generate caricatures. Although these conventional methods can generate caricature with reasonable shape exaggeration, the style of these caricatures only preserve specific styles, like black-and-white, which are monotonous and lack of details. In addition, these conventional methods mostly require multiple steps, which are too complicated for the large-scale caricature generation.

Recently, due to the success of generative adversarial networks (GANs) (Goodfellow et al., 2014) in many tasks, e.g., image generation (Yan et al., 2016; Zhang et al., 2017), image translation (Isola et al., 2017; Zhu et al., 2017a,b), image fusion (Li et al., 2020a), and face image editing (Choi et al., 2018; Xiao et al., 2018), many GAN-based methods (Cao et al., 2018; Shi et al., 2019; Zheng et al., 2019; Ding et al., 2020; Hou et al., 2020; Li et al., 2020b) for caricature generation have been introduced. As listed in the second column of Table 1, most GAN-based methods mainly decouple caricature generation into two steps, i.e., style transfer and shape exaggeration, and perform them in two separate models. The works proposed by Li et al. (2020b) and Zheng et al. (2019) performed these two steps in a single generator. CariGANs (Cao et al., 2018), WarpGAN (Shi et al., 2019), MW-GAN (Hou et al., 2020), and the work proposed by Ding et al. (2020) included a style-related and a geometric network to perform style transfer and shape exaggeration. Even though they applied two networks to simplify the process, they still suffer from some problems. Some caricatures synthesized by these methods cannot achieve the reasonable shape exaggeration to reflect the personality of the subject and even have distorted faces. For instance, WarpGAN (Shi et al., 2019) predicted control points and the corresponding displacements. However, the control points of each photo image are always the same, which fails to present the characteristics of different subjects. As illustrated in the third to fifth column of Table 1, MW-GAN (Hou et al., 2020) and CariGANs (Cao et al., 2018) use the landmark as feature to encourage the geometric network to perform personalized shape exaggeration for individual subject, but these methods not only request landmarks of photo images but also that of the caricature images. Since algorithms of the facial landmark detection are quite mature, it is easy to obtain the facial landmarks of a photo image. However, there is no any work available to detect facial landmarks for caricature. Thus, MW-GAN, CariGANs, and CariGAN can only be applicable to datasets with annotation of facial landmarks for both photos and caricatures. In addition, these GAN-based methods are easy to produce unrealistic caricature styles, which are not colorful and even completely dark. It is mainly because that these methods applied Adaptive Instance Normalization (AdaIN) (Huang and Belongie, 2017) to perform style transfer in their style network. CariGANs, MW-GAN and WarpGAN introduced AdaIN in their network to perform style transfer. However, AdaIN transfers style by scaling and shifting each feature map with adaptive parameters, which cannot learn the relationship between the caricature style and photo image and is thus not able to appropriately fuse their features. As listed in the sixth column of Table 1, the existing methods do not learn the relationship between the caricature style feature and the facial feature. Zheng et al. (2019) proposed a CyleGAN based model to achieve photo-to-caricature translation, which failed to learn the domain gap between photo and caricature and resulted in the unrealistic caricatures. CariGAN applied a Pix2Pix based generator with random noise map to transfer caricature style, which is too simple to fuse the caricature style with photo image. Even though Ding et al. (2020) employed two paired encoder-decoder networks and a contrastive style loss, the styles of the caricatures generated are not diverse.

**Table 1:** Comparison of GAN-based methods for caricature generation.

**Methods**	**Decoupling**	**Facial landmarks**		**Personalized** **shape** **exaggeration**	** Learning the** **relationship between** **style and content**	**Source code**
		**Caricature**	**Photo**			
Zheng et al. (2019)						
CariGAN (Li et al., 2020b)		✓	✓			
CariGANs (Cao et al., 2018)	✓	✓	✓	✓		
MW-GAN (Hou et al., 2020)	✓	✓	✓	✓		
WarpGAN (Shi et al., 2019)	✓					✓
Ding et al. (2020)	✓					
CariMe (Gu et al., 2021)		✓	✓	✓		✓
Ours	✓		✓	✓	✓	✓

Decoupling indicates decoupling style transfer and shape exaggeration.

To address the challenges mentioned above, we propose a novel individualized automatic caricature generation method to apply the characteristics of a subject to generate personalized caricature. Our contributions are as follow:

1. We propose a Style Attention based Global-local Aware GAN for Personalized Facial Caricature Generation, which integrates the characteristics of each subject to the network and synthesizes personalized caricature.
2. A landmark-based warp controller for personalized shape exaggeration is proposed to implement individualized image warping for caricature generation. To integrate the facial characteristics in our framework, 81 facial landmarks are employed as the control points and the displacement of these control points are predicted.
3. Moreover, to appropriately fuse the facial feature with caricature style, we introduce a style-attention module to balance the content feature of the photo image and the caricature style feature, and transfer the caricature style.
4. A multi-scale discriminators is proposed to both globally and locally discriminate the synthesized and real caricature to ensure the whole structure of synthesized caricature and the preservation of realistic details, which aims to increase the quality of the generated caricatures.

The rest of our paper is organized as follows. In Section 2, the existing methods for caricature generation are briefly reviewed. Section 3 illustrates our proposed method. Section 4 presents the experimental results and discussion, including the datasets, implementations, evaluation metrics, ablation study, and comparison with the previous methods. Finally, in Section 6, we draw conclusions.

## 2. Related works

In recent years, numerous approaches for caricature generation have been proposed. These methods can be divided into two groups. The first one, namely conventional methods (Akleman et al., 2000; Fujiwara et al., 2000; Chen et al., 2001; Liang et al., 2002; Gooch et al., 2004; Mo et al., 2004; Liu et al., 2006; Tseng et al., 2012; Yang et al., 2016), is composed of interactive methods, regularity-based methods, and learning-based methods. On the other hand, GAN-based methods (Cao et al., 2018; Shi et al., 2019; Zheng et al., 2019; Hou et al., 2020; Li et al., 2020b; Gu et al., 2021), mainly applies generative adversarial networks (GANs) (Goodfellow et al., 2014) for caricature generation.

### 2.1. Conventional methods

In interactive approaches, users can exaggerate the personalized facial features intuitively. Akleman (1997) proposed an interactive method to allow users to draw a simple template with several lines to morph original face image. However, it is only suitable for skillful users to make a recognizable caricature. A 2D deformation technique proposed by Akleman et al. (2000) generates caricature by using simplex as deformation primitives. It requires users to provide several triangle pairs to deform texture, which is also a challenge for ordinary users.The work proposed by Gooch et al. (2004) presents an approach to generate black and white illustration. The generated illustration is framed by grid line. Then, users can move the grid line of illustration intuitively to create caricatures.

The regularity-based methods automatically or semi-automatically create caricatures according to the rules summarized by researchers. Redman (1984) introduced an idea that caricature should be “exaggerating the difference from the mean” (EDFM). Most approaches follow this notion for caricature generation. Brennan (1985) first employed this notion to generate caricatures. An average face is predefined by researchers before exaggeration. During the comparison, 165 points from the subject face are mapped onto corresponding points on the average face. The distance between each pair of corresponding points on two faces is represented as vector. Then, the subject face can be exaggerated by multiplying each vector by a rate of exaggeration. This rule is widely used in many regularity-based methods (Pujol et al., 2000; Chiang et al., 2004; Mo et al., 2004; Lai et al., 2006). Nevertheless, there is limitation in this rule. Mo et al. (2004) claimed that EDFM might not create the best caricature, since it only consider the difference from the mean. Besides the average face, a standard face is also used as reference face in some approaches (Gooch et al., 2004; Ni et al., 2008). Given an input face image, Ni et al. (2008) proposed to evaluate the differences between the input face and the standard face and then the distinctiveness of input image can be computed.

The learning-based approaches utilize machine learning techniques to solve this problem. These approaches require a large and paired training dataset. Each pair contains an original face image and a corresponding caricature image drawn by artist. Liu et al. (2006) proposed to adopt PCA (Principal Components Analysis) to obtain the principal components of facial features. Then, SVR (Support Vector Regression) is utilized to learn the mapping between the principal component space of original face image and that of caricature image. An example-based method proposed by Liang et al. (2002) first decoupled the process of caricature generation into two parts, i.e., shape exaggeration and texture style transferring. The shape exaggeration used PLS (Partial Least Square) to classify the face images pairs into several prototypes and then predict the facial features to be exaggerated and the rate of exaggeration. These works (Liu et al., 2006; Yang et al., 2016) mainly apply linear method to estimate target caricature, while others employed the non-linear approaches. The works proposed by Shet et al. (2005) and Lai et al. (2006) introduced neural networks to capture the style of the real artists.

Although these conventional methods can generate caricature with reasonable shape exaggeration, the caricature synthesized by these methods can only preserve specific styles, like black and white, which are quite monotonous and lack of details. In addition to this, these methods are too complicated for the large-scale caricature generation, since they involve multiple steps.

### 2.2. GAN-based methods

Due to the substantial progress of generative adversarial networks (GANs) (Goodfellow et al., 2014), it has been widely used in many tasks, e.g., image generation (Yan et al., 2016; Zhang et al., 2017; Xian et al., 2018), image translation (Isola et al., 2017; Kim et al., 2017; Yi et al., 2017; Zhu et al., 2017a,b; Emami et al., 2020), image fusion (Li et al., 2020a), and face image editing (Li et al., 2011; Choi et al., 2018; Natsume et al., 2018; Xiao et al., 2018; Yan et al., 2019; Wang et al., 2020; Zhang and Ling, 2020). A number of GAN-based caricature generation works have also been available in literature.

These GAN-based methods mainly decouple the process of caricature generation into two steps, i.e., style transfer and shape exaggeration. The works proposed by Li et al. (2020b) and Zheng et al. (2019) performed these two steps in a single generator. Zheng et al. (Zheng et al., 2019) introduced CycleGAN (Zhu et al., 2017a) like framework to perform photo-to-caricature translation and distinguish fake and real caricatures with a coarse discriminator and a fine discriminator. Li et al. (2020b) proposed a weakly paired adversarial learning, namely CariGAN for caricature generation. It first fed the concatenation of noise, face image and facial mask to the U-Net generator and then utilized the image fusion mechanism to get the results. Other methods (Cao et al., 2018; Shi et al., 2019; Hou et al., 2020) mainly applied a style network and a geometric network to decouple the process into two steps. CariGANs (Cao et al., 2018) proposed a CariStyGAN for style transfer and CariGeoGAN for shape exaggeration. CariStyGAN applied the structure of MUNIT (Huang et al., 2018) to perform photo-to-caricature style transfer. To learn the geometry exaggeration, CariGeoGAN applied PCA on the landmarks of the input face image and caricature image. Then, it used a CycleGAN like framework to learn the mapping between the PCA component of input face image and that of caricature image. WarpGAN (Shi et al., 2019) proposed a deformable generator to automatically transfer style and exaggerate texture image. As for style transfer, it encoded the style and content with two separate encoders. To impose a random style vector on content feature, a decoder with adaptive instance normalization is applied in this process. Additionally, it introduced a warp controller to predict the control points and their offsets. The final caricature is generated by warping rendered texture according to the source and target control points. Hou et al. (2020) proposed a MW-GAN with a style network and a geometric network to generate caricature with various styles. In geometric network, MW-GAN applied the landmarks to perform shape exaggeration. Ding et al. (2020) proposed an unsupervised constrastive photo-to-caricature translation architecture, which includes two paired encoder-decoder networks and distortion prediction module to achieve style transfer and shape exaggeration. CariMe (Gu et al., 2021) proposes a multi-exaggeration warper network to learn the distribution-level mapping from photos to facial exaggerations and a styler to transfer the caricature style to the warped photo.

When performing reasonable shape exaggeration, these GAN-based methods suffer from different problems. As for WarpGAN, the predicted control points are always the same, which brings similar shape exaggeration for each subject. As illustrated in third to fifth column of Table 1, both CariGANs and MW-GAN applied the landmarks of each subject to encourage the geometric network to perform characteristic shape exaggeration, but these methods require not only the landmarks of photo images but also that of the caricature images. Since the facial landmarks detection algorithm are quite mature, the facial landmarks of a photo image are easy to obtain. However, there is no any algorithm available for caricature facial landmarks detection. Thus, both MW-GAN and CariGANs can only be applied to datasets with facial landmarks annotation of both photos and caricatures. There is still a research gap of how to perform characteristic shape exaggeration without requirement of caricature facial landmarks. In this paper, we propose a landmark-based warp controller for personalized shape exaggeration to implement individualized image warping, which only requests the landmarks of photo images.

In addition, these GAN-based methods are easy to produce bad cases, which are not realistic due to the low contrast. Adaptive Instance Normalization (AdaIN) (Huang and Belongie, 2017) in their style networks is the main cause of such artifacts. AdaIN scales and shifts each feature map with adaptive parameters, which cannot learn the relationship between the caricature style and photo images and balance the features of photo images and caricature style. As listed in the sixth column of Table 1, the existing methods do not learn the relationship between the caricature style feature and the facial feature. Even though Ding et al. (2020) proposed two paired encoder-decoder network to learn the common content space of photo and caricatures, the styles of caricatures generated by their method suffer from artifacts and are not diverse. Thus, there is a research gap of how to learn the relationship between the caricature style and photo images and perform reasonable caricature style transfer. In this paper, we introduce a style-attention module to tackle this issue and a multi-scale discriminator to increase the quality of the generated caricature.

## 3. Methods

### 3.1. Overview of network architecture

**Figure 2:**
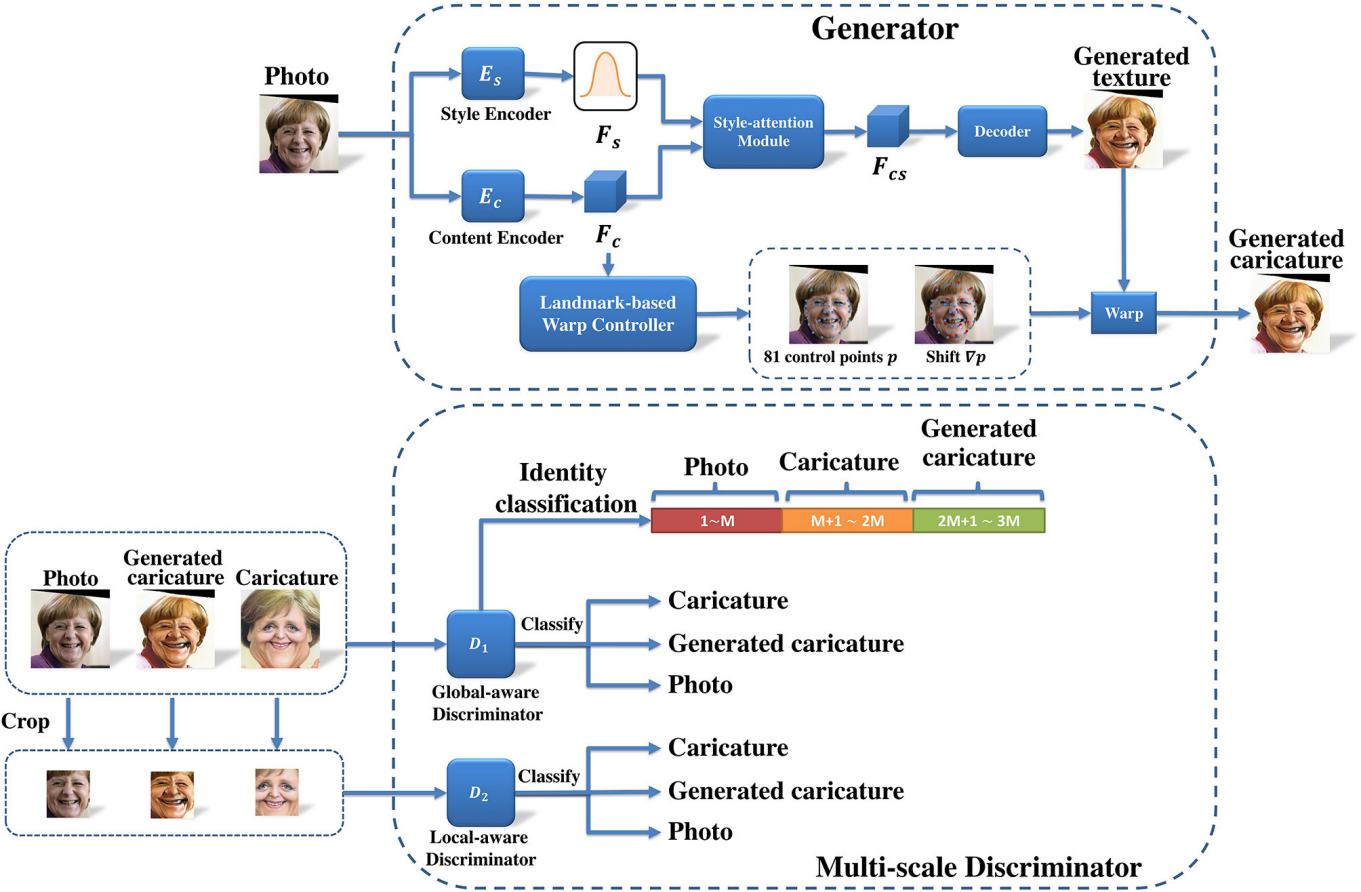
The network architecture of the proposed framework, including a generator with style-attention module and landmark-based warp controller for personalized shape exaggeration, and a multi-scale discriminator.

Figure 2 illustrates the framework of our proposed method for individualized automatic caricature generation. In this work, we decouple the caricature generation into two processes, i.e., caricature style transfer and shape exaggeration. As for caricature style transfer, we employ a style-attention module to transfer the caricature style. Regarding to shape exaggeration, a landmark-based warp controller for personalized shape exaggeration is used to perform individualized image warping.

Given a 2D photo image *x* $\left(x\in {\mathbb{R}}^{H\times W\times C},x\in X\right)$, we first apply style encoder and content encoder to output style feature *F*_*s*_ = *E*_*s*_(*x*) and content feature *F*_*c*_ = *E*_*c*_(*x*), respectively. Here, *H*, *W*, and *C* are height, width and number of channels respectively. Then, style-attention module fuses *F*_*c*_ and a random latent style code $F_{s}^{\text{′}}\sim N(0,I)$ to render the caricature style to content feature and obtains a stylized content feature *F*_*cs*_. Afterwards, the decoder takes *F*_*cs*_ to generate a caricature texture *R*(*F*_*cs*_). These processes can stylize the photo image with caricature style. To further exaggerate the caricature texture, we utilize a landmark-based warp controller for personalized shape exaggeration to predict the control points and the corresponding displacements to warp the generated texture.

To provide more details about our method, the architecture of style encoder, content encoder, decoder, local-aware discriminator and global-aware discriminator are shown in Table 2. We list the output size and detailed information for each layer, which includes the kernel size, the number of filter, the size of stride, the type of normalization and the activation function. The abbreviations in the table include: IN, instance normalization; BN, batch normalization; LN, layer normalization; LReLU, Leaky ReLU with slope 0.2; M, the number of the identity.

**Table 2:** The architecture of style encoder, content encoder, decoder, local-aware discriminators, and global-aware discriminators.

**Layer name**	**Output size**	**Layer information**
**Style encoder**		
Conv1	256 × 256	7 × 7, 64, LN, ReLU
Conv2	128 × 128,	4 × 4, 128, stride 2, LN, ReLU
Conv3	64 × 64	4 × 4, 256, stride 2, LN, ReLU
**Content encoder**		
Conv1	256 × 256	7 × 7, 64, LN, ReLU
Conv2	128 × 128	4 × 4, 128, stride 2, LN, ReLU
Conv3	64 × 64	4 × 4, 256, stride 2, LN, ReLU
Residual block	64 × 64	$\left[\begin{array}{c} 3\times 3,256\\ 3\times 3,256 \end{array}\right]\times 3$, IN, ReLU
**Decoder**		
Residual block	64 × 64	$\left[\begin{array}{c} 3\times 3,256\\ 3\times 3,256 \end{array}\right]\times 3$, IN, ReLU
Upscale1	128 × 128	Bilinear interpolation × 2
Deconv1	128 × 128	5 × 5, 128, LN, ReLU
Upscale2	256 × 256	Bilinear interpolation × 2
Deconv2	256 × 256	5 × 5, 64, LN, ReLU
Conv1	256 × 256	7 × 7, 3
Output	256 × 256	Tanh
**Local-aware discriminator**		
Conv1	56 × 48	4 × 4, 32, stride 2, BN, LReLU
Conv2	28 × 24	4 × 4, 64, stride 2, BN, LReLU
Conv3	14 × 12	4 × 4,128, stride 2, BN, LReLU
Conv4	7 × 6	4 × 4, 256, stride 2, BN, LReLU
Output layer ($D_{2}^{adv}$)	7 × 6	3 × 3, 3
**Global-aware discriminator**		
Conv1	128 × 128	4 × 4, 32, stride 2, BN, LReLU
Conv2	64 × 64	4 × 4, 64, stride 2, BN, LReLU
Conv3	32 × 32	4 × 4, 128, stride 2, BN, LReLU
Conv4	16 × 16	4 × 4, 256, stride 2, BN, LReLU
Conv5	8 × 8	4 × 4, 512, stride 2, BN, LReLU
Flatten	(8 × 8 × 512) × 1	Flatten
Output layer ($D_{1}^{idt}$)	3*M*	Fully connected layer
Output layer ($D_{1}^{adv}$)	8 × 8 × 3	3 × 3, 3

### 3.2. Style-attention module

When transferring the caricature style to a photo image, most deep learning based methods applied the Adaptive Instance Normalization (AdaIN) (Huang and Belongie, 2017) and MUNIT (Huang et al., 2018) to transfer the style. The approaches (Shi et al., 2019) using AdaIN simply adjusted the mean and variance of the content feature to match those of the style code. Even though it is effective to transfer the caricature style, the synthesized caricature texture suffers from quality problem. Moreover, stylized photo images generated by these methods always preserve low saturation, which sometimes failed to balance the latent style code and content feature. CariGANs (Cao et al., 2018) employed MUNIT to perform caricature style transfer, which needs a deep network and a set of complicated losses. Thus, we need a novel method to achieve personalized caricature style transfer with less parameters and losses.

**Figure 3:**
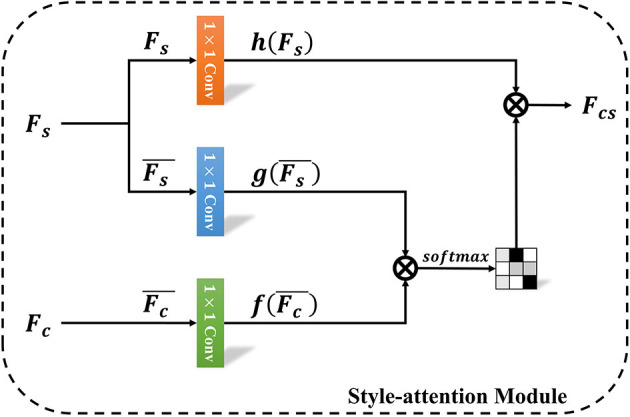
The overview of style-attention module.

Inspired by the method proposed by Park and Lee (2019), we propose a style-attention module (SAM) to perform personalized caricature style transfer with less parameter. When it comes to the difference, Park and Lee (2019) applied SANet to perform style transfer between the style image and content image, while we use style-attention module to stylize the content feature with the latent style feature *F*_*s*_. The goal of style-attention module is to balance the latent style feature and content feature. As demonstrated in Figure 3, it first employs a 1 × 1 convolutional layer to output a weighted style feature *h*(*F*_*s*_). Then, both the style and content feature are normalized and weighted by two separate 1 × 1 convolutional layers (*g, f*). Finally, the stylized content feature are computed as follows:


(1) $$\begin{array}{l} F_{cs}=softmax\left(f{\left(\bar{F_{c}}\right)}^{T}g\left(\bar{F_{s}}\right)\right)h\left(F_{s}\right) \end{array}$$

where $\bar{F}$ denotes a mean-variance channel-wise normalized version of *F*. To simplify the representation, the style-attention module can be defined as:


(2) $$\begin{array}{l} F_{cs}=SAM\left(F_{c},F_{s}\right) \end{array}$$

This module can locally embed the latent style feature in each position of the content feature by mapping a relationship between the style and content feature maps, which is the output of softmax in this module.

To further generate a caricature texture, a decoder *R* takes the stylized content feature *F*_*cs*_ and outputs a generated texture *R*(*F*_*cs*_). Moreover, we introduce two reconstruction losses to supervise the encoders to correctly and effectively extract style feature and content feature. The reconstruction losses are formulated as:


(3) $$\begin{array}{lll} L_{rec}^{p} & = & E_{x\in X}\left[{\left||R\left(SAM\left(E_{c}\left(x\right),E_{s}\left(x\right)\right)\right)-x|\right|}_{1}\right], \end{array}$$

(4) $$\begin{array}{lll} L_{rec}^{c} & = & E_{y\in Y}\left[{\left||R\left(SAM\left(E_{c}\left(y\right),E_{s}\left(y\right)\right)\right)-y|\right|}_{1}\right] \end{array}$$

where *y* denotes a real caricature image.

### 3.3. Landmark-based warp controller for personalized shape exaggeration

To automatically perform shape exaggeration, WarpGAN (Shi et al., 2019) applied a warp controller to predict the control points and the corresponding displacements. However, the predicted control points are mostly at the same position, which cannot present the differences among individuals. Even though CariGANs (Cao et al., 2018) achieved individual shape exaggeration, by using an additional CariGeoGAN to predict the exaggerated control points embedded by PCA, it requires the landmarks of caricatures.

To achieve personalized shape exaggeration, CariGANs (Cao et al., 2018) and MW-GAN (Hou et al., 2020) applied landmarks to learn the individualized deformation for each subject. However, these methods needs the facial landmarks of both photo images and caricature images. Unfortunately, current state of the art landmark detections are not able to achieve accurate results on caricature images. Thus, we introduce a landmark-based warp controller for personalized shape exaggeration to perform individualized image warping, without requirement of facial landmarks of caricature images.

**Figure 4:**
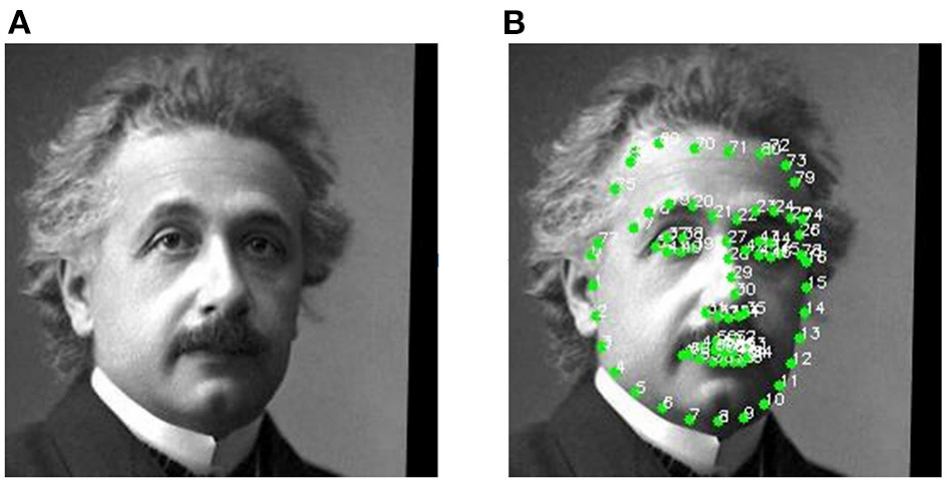
The photo samples for 81 facial landmarks detection. **(A)** The original photo and **(B)** the photo with 81 landmarks.

As demonstrated in Figure 4, we employ 81 facial landmarks as control points *p*. The landmark-based warp controller consists of two fully connected layers. It predicts the displacement Δ*p* = {Δ*p*_1_, Δ*p*_2_, ..., Δ*p*_81_} for each control point *p* = {*p*_1_, *p*_2_, ..., *p*_81_}, where *p*_*i*_ and Δ*p*_*i*_ is a 2D vector in the u-v space. Afterwards, these points are fed into a differentiable warping module (Cole et al., 2017). The destination points are $p^{\prime }=\{p_{1}^{\prime },p_{2}^{\prime },...,p_{81}^{\prime }\}$, where $p_{i}^{\prime }=p_{i}+\Delta p_{i}$. We compute a *H* × *W* grid sampler *via* thin-plate spline interpolation. Then, we generate the warped image through bi-linear sampling (Jaderberg et al., 2015). The final generated caricature can be represented as:


(5) $$G(x,s)=Warp(R(SAM(E_{c}(x),F_{s}^{\prime })),p,\Delta p),$$

Since the warping module (Cole et al., 2017) is differentiable, our network can be trained in an end-to-end manner.

### 3.4. Multi-scale discriminator

Similar to most methods, we adopt the patchGAN (Isola et al., 2017) as our discriminator. However, taking the whole image as input can only guide the generator to generate caricatures with correct global structure. Thus, we further introduce both global-aware discriminator *D*_1_ and local-aware discriminator *D*_2_ to ensure both correct structure and realistic details.

#### 3.4.1. Global-aware discriminator

##### 3.4.1.1. Adversarial loss

The global-aware discriminator is trained as a 3-class classifier to discriminate the difference between the real caricatures, the generated caricatures and photos. Let $D_{1}^{1},D_{1}^{2}$, and $D_{1}^{3}$ represent the logits for the three classes of photo, real caricature, and generated caricature, respectively, the global-aware adversarial loss is defined as :


(6) $$L_{G}^{global}=-E_{x\in X,F_{s}^{\prime }\in S}\left[logD_{1}^{1}(G(x,F_{s}^{\prime }))\right]$$

(7) $$\begin{array}{l} L_{D_{1}}^{adv}=-E_{y\in Y}\left[logD_{1}^{1}(y)\right]-E_{x\in X}\left[logD_{1}^{2}(x)\right]\\ \;-E_{x\in X,F_{s}^{\prime }\in S}\left[logD_{1}^{3}(G(x,F_{s}^{\prime }))\right] \end{array}$$

##### 3.4.1.2. Identity preserving loss

Even though the patchGAN based discriminator can ensure quality of generated caricatures, it still fails to preserve the difference among different subjects. To simultaneously preserve the identities of photos and ensure the quality of style, we also train discriminator as a 3M-classifier, which is proposed in WarpGAN (Shi et al., 2019) and *M* is the number of identities. The identities of photos, real caricatures and generated caricatures correspond to the first, second and third *M* classes, respectively. Let *l*^*p*^, *l*^*c*^/*in*{1, 2, 3, ..., *M*} be the identity label for photos and caricatures. The identity preserving losses for generator and discriminator are formulated as :


(8) $$L_{G}^{idt}=-E_{x\in X,F_{s}^{\prime }\in S}\left[logD_{1}(l^{c};G(x,F_{s}^{\prime }))\right]$$

(9) $$\begin{array}{l} L_{D_{1}}^{idt}=-E_{y\in Y}\left[logD_{1}(l^{c};y)\right]-E_{x\in X}\left[logD_{1}(l^{p}+M;x)\right]\\ \;-E_{x\in X,F_{s}^{\prime }\in S}\left[logD_{1}(l^{p}+2M;G(x,F_{s}^{\prime }))\right] \end{array}$$

#### 3.4.2. Local-aware discriminator

Although the global-aware discriminator can ensure the global structure and style, some details of fake caricature are always overlooked. In addition, the receptive field of global-aware discriminator is 158 × 158, which are too large to focus on details. Thus, we further introduce a local-aware discriminator with smaller receptive field to ensure the fidelity of fake caricature and preserve the identity of photos. Similar to the global-aware discriminator, the local-aware discriminator is also trained as a 3-class classifier, whose receptive field is 78 × 78. We first center crop a patch of size 96 × 112 from photos, fake caricature and real caricature, respectively. Then, the cropped images are fed into local-aware discriminator to distinguish the three types. The local-aware adversarial loss is defined as follows:


(10) $$L_{G}^{local}=-E_{x\in X,F_{s}^{\prime }\in S}\left[logD_{1}^{1}(Crop(G(x,F_{s}^{\prime })))\right]$$

(11) $$\begin{array}{l} L_{D_{2}}^{adv}=-E_{y\in Y}\left[logD_{2}^{1}(Crop(y))\right]\\ \;-E_{x\in X}\left[logD_{2}^{2}(Crop(x))\right]\\ \;-E_{x\in X,F_{s}^{\prime }\in S}\left[logD_{2}^{3}(Crop(G(x,F_{s}^{\prime })))\right] \end{array}$$

where $D_{2}^{1},D_{2}^{2}$, and $D_{2}^{3}$ represent the logits for the three classes of caricatures, photos and generated images, and *Crop*(*x*) denotes the center crop operation.

### 3.5. The full objective functions

The overall objective functions for generator and discriminator are as follows:


(12) $$\begin{array}{l} \begin{array}{cc} \underset{G}{\min}L_{G}= & \lambda_{1}L_{G}^{global}+\lambda_{2}L_{G}^{local}+\lambda_{3}L_{G}^{idt}\\ +\lambda_{4}\left(L_{rec}^{p}+L_{rec}^{c}\right) \end{array} \end{array}$$

(13) $$\begin{array}{l} \underset{D}{\min}L_{D}=\lambda_{1}L_{D_{1}}+\lambda_{2}L_{D_{2}} \end{array}$$

where λ_1_, λ_2_, λ_3_, and λ_4_ denote the hyper-parameters to balance different losses.

## 4. Experiments and discussion

### 4.1. Datasets

We conduct our experiments on two publicly available datasets, i.e., the WebCaricature dataset (Huo et al., 2017) and the CaVINet dataset (Garg et al., 2018).

The **WebCaricature** dataset consists of 5,974 photos and 6,042 caricatures from 252 identities. For each caricature and photo, 17 facial landmarks are provided. All the images are aligned with similarity transformation using five landmarks and resized to 256 × 256. We randomly split the dataset into two parts, i.e., training set and testset. The training set contains 3,016 photos and 3,112 caricatures from 126 identities. The testset includes 2,958 photos and 2,930 caricatures from 126 identities.

The **CaVINet** dataset contains 5,091 caricatures and 6,427 photos. The number of both caricatures and photos for each identity ranges from 10 to 15. These images are from 205 identities. We first align all the photos with similarity transformation using five landmarks detected by Dlib (King, 2009) and then resize them to 256 × 256. Note that we do not detect the landmarks for caricature and align the caricature images. We randomly split the dataset into a training set of 103 identities (3,117 photos and 2,562 caricatures) and a test set of 102 identities (2,817 photos and 2,227 caricatures).

Since our proposed method requires facial landmarks for all the photo images, we prepare 81 facial landmarks for both WebCaricature and CaVINet datasets. We first apply Dlib (King, 2009) to extract 68 facial landmarks, which includes the contours for face, mouse, eyes, eye brows and nose. Afterwards, we create the Surrey Face Model (Huber et al., 2016) and extract the coordinates around the forehead, which are the corners of the triangles making up the mesh. The extracted 81 facial landmarks are demonstrated in Figure 4. All the testing images in this paper are from the identities of testing set.

### 4.2. Implementation

#### 4.2.1. Experiments settings

Our models are optimized with Adam (Kingma and Ba, 2014), where β_1_ = 0.5 and β_2_ = 0.9. Each batch is composed of a random pair of a photo image and a caricature image. We train the models for 100,000 steps. We train generator and discriminator alternatively. The learning rate is 0.0001 for the first 50,000 steps and linearly decays to 0 over the next 50,000 steps. We empirically set λ_1_ = 2, λ_2_ = 1, λ_3_ = 1, and λ_4_ = 10. We conduct our experiments using Tensorflow r1.12 and one Tesla P100 NVIDIA GPU.

### 4.3. Evaluation metrics

As aforementioned, caricatures should contain three main elements, i.e., likeness, exaggeration, and style. To evaluate the first characteristic, we perform face identification to evaluate whether generated caricatures have the same identity as photos. Besides, we apply Fréchet Inception Distance (FID) (Heusel et al., 2017) to measure whether the caricature style and shape exaggeration of generated caricatures share the same distribution as that of real caricatures.

For **face identification**, we introduce Cumulative Match Curve (CMC) (Bolle et al., 2005) to measure the performance. Each of the generated caricatures from prob set is compared against the real photos from the gallery set and the top *k* pairs with the smallest distance were identified. To measure the distance of two images, we first adopt the network proposed by Wen et al. (2016) to extract 512-dimension identity features from fake caricatures and real photos and then apply the cosine distance to measure the similarity. An identification is decided as correct if the subject of the generated caricature was included in the top *k* list. All identification experiments are performed to report both CMC and the rank one-to-ten (*k* ∈ {1, ..., 10}) matching number of the generated caricatures. To construct a pair of gallery set and prob set for WebCaricature datset, we randomly pick 126 identities from testset and employ the corresponding 126 photos as gallery set. Then, we generate the caricatures from the rest of 2, 832 = 2, 958 − 126 photos as the prob set. Finally, we randomly construct 10 pairs of gallery set and prob set and compute the average for the results.

The **Fréchet Inception Distance (FID)** is introduced to measure the distribution of the fake caricatures generated by our proposed model and the real caricatures. The lower FID means that the distribution of synthesized caricatures are closer to that of real caricatures. As for the WebCaricature dataset, we randomly pick 2,930 photos of testset and generate the corresponding caricatures by our proposed model. Afterwards, we measure the FID between the generated caricatures and all the real caricatures from testset. Similarly, to evaluate on the CaVINet dataset, we generate 2,817 caricatures from the photos of testset and compute the FID between the fake and real caricatures.

### 4.4. Ablation study

We conduct ablation study on the WebCaricature dataset to evaluate the effectiveness of each module, i.e., style-attention module (SAM), landmark-based warp controller for personalized shape exaggeration (LWC), and multi-scale discriminator (MD). We adopt WarpGAN as our baseline. When ablating style-attention module, we replace the instance normalization of decoder to Adaptive Instance Normalization (AdaIN) and require style encoder to predict the corresponding β and γ. As for the landmark-based warp controller, we applied the warp controller of WarpGAN for ablation study. When performing ablation study on multi-scale discriminator, we simply remove the local-aware discriminator. The ablation study results are shown in Table 3. When the multi-scale discriminator is adopted, the FID is improved by 1.22. Moreover, when integrating the style-attention module, the FID is decreased by 1.42 over the baseline. Furthermore, when the landmark-based warp controller is employed, the FID is improved by 1.83. If we integrate both multi-scale discriminator and style-attention module to baseline, FID is remarkably declined by 3.03 over the baseline. Finally, the proposed method integrated with multi-scale discriminator, style-attention module and the landmark-based warp controller achieves the best performance for caricature generation with a FID of 32.62.

**Table 3:** The ablation study results on the WebCaricature dataset.

**Combination**			**FID**
**MD**	**SAM**	**LWC**	
			36.28
✓			35.06
	✓		34.86
		✓	34.45
✓	✓		33.25
✓	✓	✓	**32.62**

The abbreviation for multi-scale discriminator, style-attention module, and landmark-based warp controller for personalized shape exaggeration are SAM, LWC, and MD, respectively. The values in bold indicate the best performance compared with other methods.

### 4.5. Comparison with previous methods

In this section, we qualitatively and quantitatively evaluate our proposed method on both the WebCaricature and CaVINet datasets. We mainly compare with the GAN-based image translation methods, including CycleGAN (Zhu et al., 2017a) and MUNIT (Huang et al., 2018), and caricature generation methods, i.e., WarpGAN (Shi et al., 2019) and CariMe (Gu et al., 2021). The reason why we choose WarpGAN (Shi et al., 2019) as the representative method for caricature generation is that it does not require the annotation of facial landmarks for caricature images, which is under the same settings as our method. Also, among the existing methods, it is the only one that released the source code.

#### 4.5.1. Qualitative evaluation

**Figure 5:**
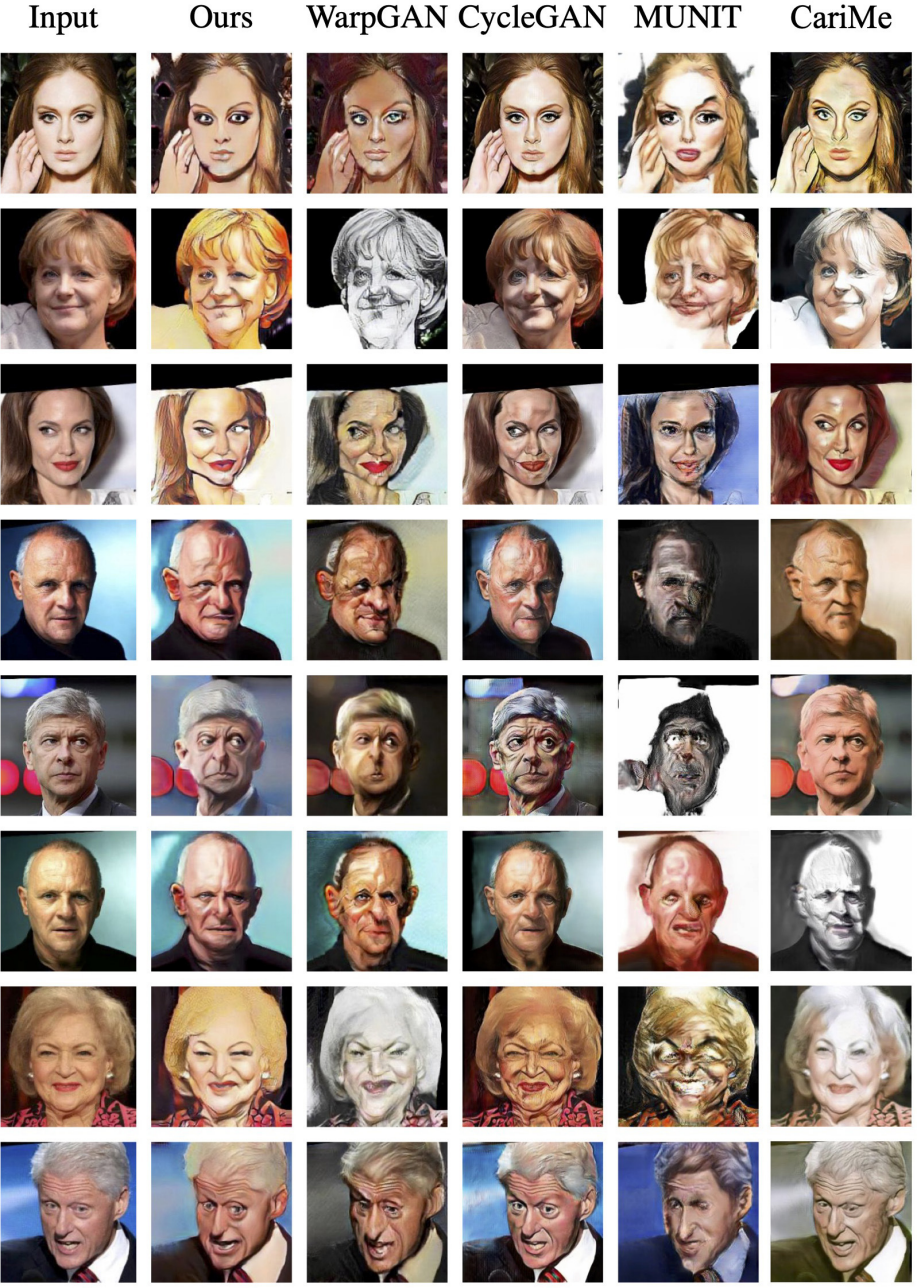
The visual results for different methods on WebCaricature dataset.

**Figure 6:**
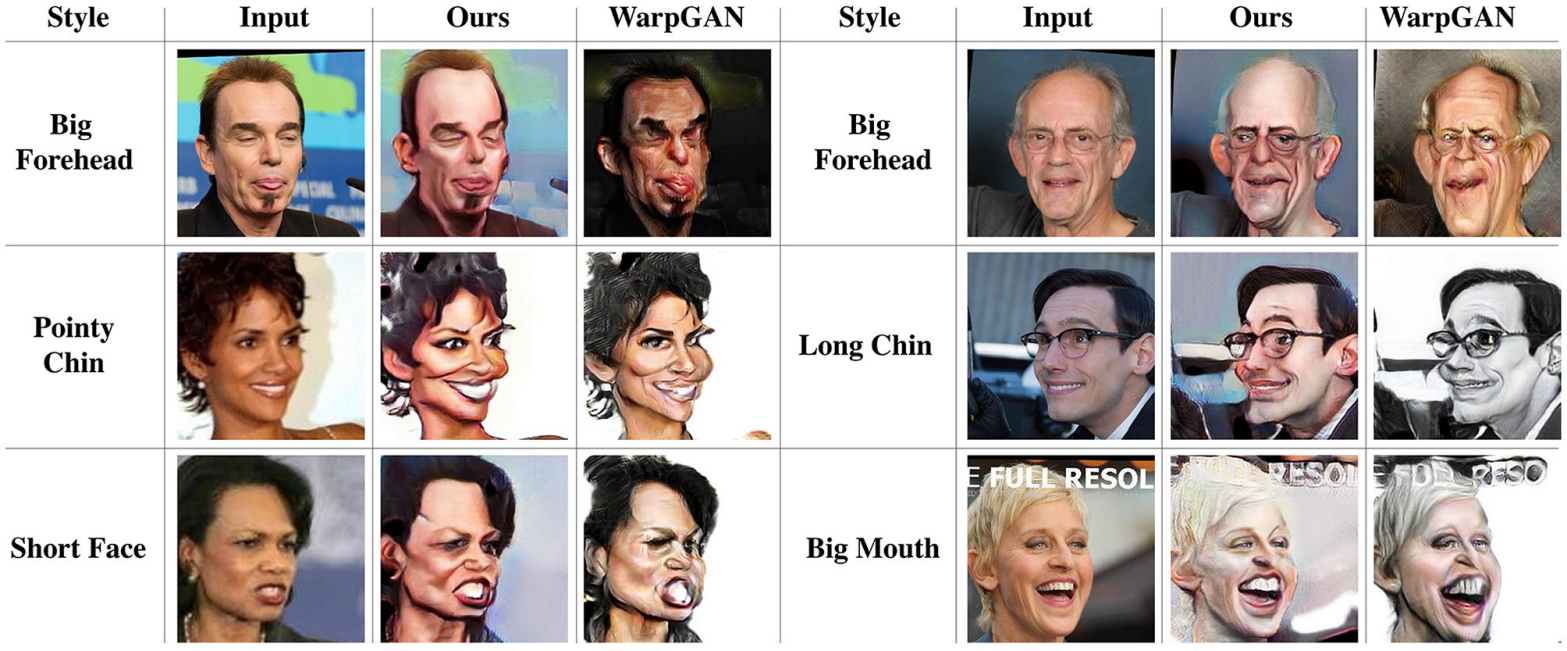
The typical exaggeration styles learned by our proposed method and WarpGAN.

As for the WebCaricature dataset, Figure 5 demonstrates the caricatures generated by our proposed method, WarpGAN (Shi et al., 2019), CariMe (Gu et al., 2021), CycleGAN (Zhu et al., 2017a), and MUNIT (Huang et al., 2018). As visualized in the figure, our proposed method achieves much better performance than the existing methods. The style of the caricatures generated by WarpGAN (Shi et al., 2019), CycleGAN (Zhu et al., 2017a), and MUNIT (Huang et al., 2018) are dark and not colorful enough, which are not as natural as the style of caricaturist. Furthermore, the caricatures synthesized by CycleGAN (Zhu et al., 2017a) are almost the same as the input photos, which does not perform style transfer and shape exaggeration. Furthermore, we compare some typical exaggeration styles generated by our proposed method and WarpGAN (Shi et al., 2019). As shown in Figure 6, our proposed method achieves better performance in the big forehead style generation. As for the long chin style, the caricature generated by our proposed method are much more significant than that generated by WarpGAN. Thus, compared to WarpGAN (Shi et al., 2019), our proposed method can present more reasonable and personalized shape exaggeration.

**Figure 7:**
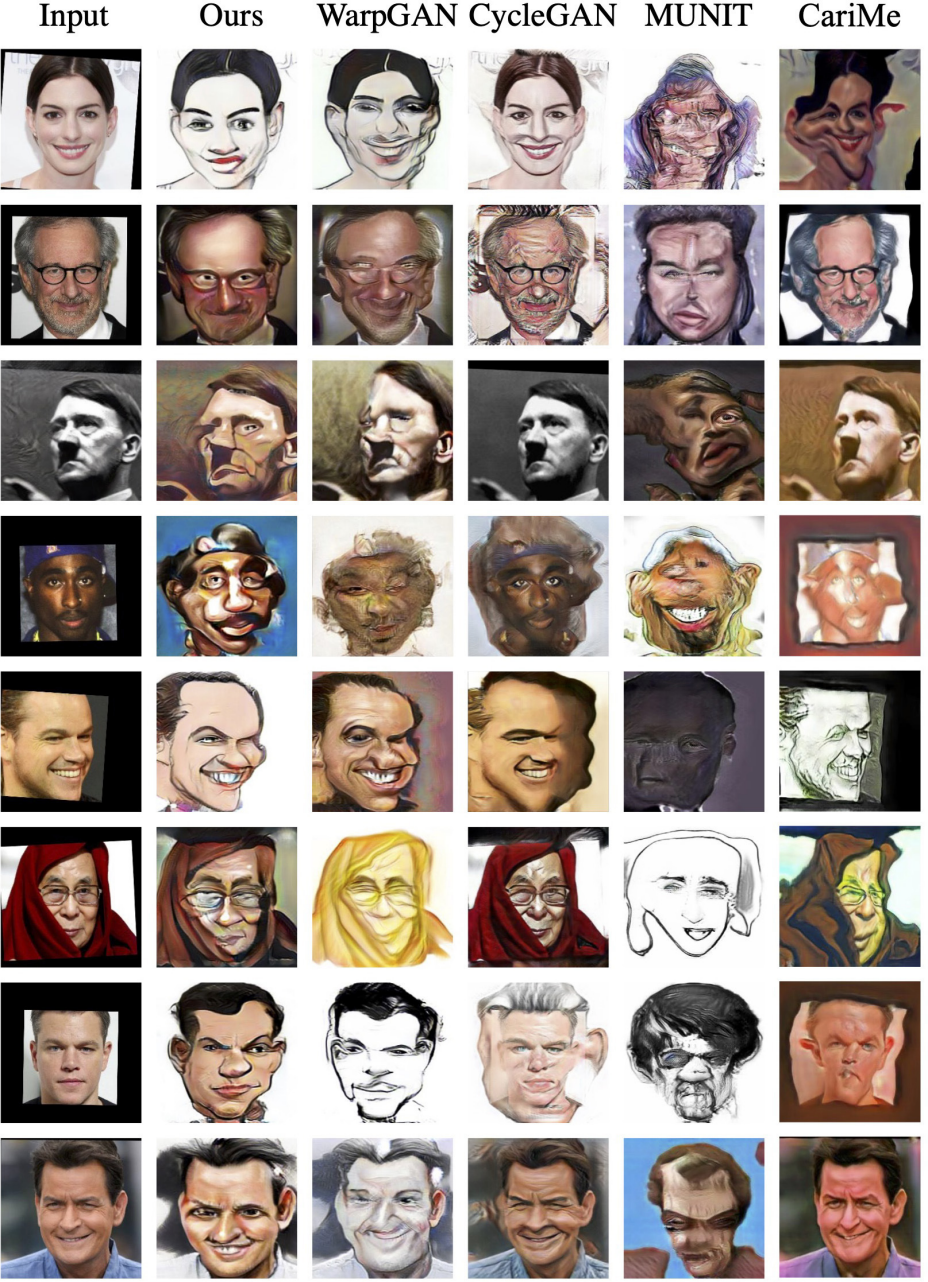
The visual results for different methods on CaVINet dataset.

As for the CaVINet dataset, Figure 7 displays the caricatures generated by our proposed method, WarpGAN (Shi et al., 2019), CariMe (Gu et al., 2021), CycleGAN (Zhu et al., 2017a), and MUNIT (Huang et al., 2018). Apparently, the caricatures generated by other methods are not as colorful as those generated by our model. In addition, these methods do not perform reasonable shape exaggeration. Similar to the WebCaricature dataset, it seems that CycleGAN (Zhu et al., 2017a) still does not perform style transferring and shape exaggeration. Moreover, compared to these methods, our proposed method can better preserve the identities of the input photos. Finally, the caricatures synthesized by our proposed method are more realistic than those generated by other methods.

#### 4.5.2. Quantitative evaluation

To evaluate different methods quantitatively, we first employ FID as our evaluation metrics. Then, we perform a user study on the visual quality of the caricatures generated by our proposed method, WarpGAN (Shi et al., 2019), CycleGAN (Zhu et al., 2017a), and MUNIT (Huang et al., 2018). In addition, we also quantify the identity preservation accuracy for caricatures generated by our proposed method and WarpGAN (Shi et al., 2019).

Table 4 lists the FID of the caricatures generated by different methods. Our proposed method achieves the lowest FID for caricature generation on both WebCaricature and CaVINet datasets, which suggests that it synthesizes more realistic and natural caricature than the other methods.

**Table 4:** The quantitative results (FID) of existing methods on the WebCaricature and CaVINet dataset.

**Methods**	**WebCaricature**	**CaVINet**
CycleGAN (Zhu et al., 2017a)	42.24	46.76
MUNIT (Huang et al., 2018)	50.01	45.45
WarpGAN (Shi et al., 2019)	36.28	41.45
CariMe (Gu et al., 2021)	33.56	40.29
Ours	**32.62**	**38.47**

The values in bold indicate the best performance compared with other methods.

As for user study, we apply the four approaches to generate caricatures from 50 photos on WebCaricature and CaVINet dataset, respectively. The 400 = 50 × 4 × 2 generated caricatures are shown to the volunteers and they are asked to select the best caricature, in terms of overall visual quality. In addition, they are also asked to select their favorite caricatures in terms of either style transferring or shape exaggeration. As a number of 22 volunteers participated the questionnaire, a maximum of 6, 600 = 100 × 3 × 22 votes can be received for each approach. Table 5 demonstrates the ratio of votes received for each model. The caricatures generated by our proposed method receive about half of the total votes in both WebCaricature and CaVINet datasets and rank the first place out of the three methods. It suggests that our proposed method achieves the best performance in the perceptual study.

**Table 5:** The perception evaluation of different models on WebCaricature and CaVINet datasets.

	**WebCaricature**			**CaVINet**		
	**Overall**	**Style transfer**	**Shape exaggeration**	**Overall**	**Style transfer**	**Shape exaggeration**
MUNIT (Huang et al., 2018)	1.00%	1.45%	11.27%	0.55%	0.82%	7.91%
CycleGAN (Zhu et al., 2017a)	20.55%	24.09%	10.55%	12.36%	12.00%	4.55%
WarpGAN (Shi et al., 2019)	20.00%	25.55%	26.82%	14.73%	17.73%	26.64%
Ours	**58.45%**	**48.91%**	**51.36%**	**72.36%**	**69.45%**	**60.91%**

The values in bold indicate the best performance compared with other methods.

**Figure 8:**
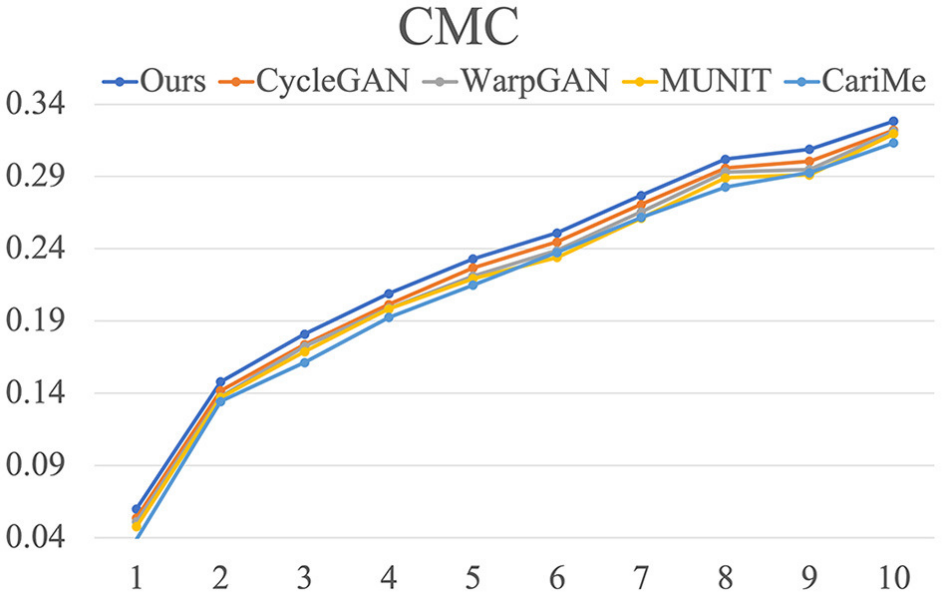
The CMC results for our proposed method and existing methods on WebCaricature dataset.

As for identity preservation, we employ CMC and the rank one-to-ten matching number of the generated caricatures to quantify the performance of face identification. Figure 8 visualizes the results for our proposed method and existing methods on WebCaricature dataset. From rank 1 to 10, our proposed method achieves higher matching rates than WarpGAN (Shi et al., 2019). It indicates that our proposed method can preserve identities much better than WarpGAN (Shi et al., 2019). As illustrated in Table 6, our proposed method achieves larger matching numbers than WarpGAN (Shi et al., 2019), from rank 1 to 10. It suggests that our proposed method perform better in preserving identities.

**Table 6:** The rank 1-to-10 matching numbers for our model and WarpGAN on the WebCaricature dataset.

**Rank**	**WarpGAN (Shi et al., 2019)**	**Ours**
1	271.0	288.8
2	397.1	414.4
3	490.5	511.6
4	573.8	589.4
5	647.2	658.5
6	711.6	723.7
7	767.6	779.7
8	819.8	834.0
9	868.5	887.1
10	917.8	932.1

As shown in Table 7, we compare the resource demand of our method and existing methods. Compared with most of methods, our proposed method requires less computation resource with 26.2 × 10^6^ parameters, ranking the second place after WarpGAN (Shi et al., 2019).

**Table 7:** The resource demand of existing methods.

**Methods**	**FLOPs (10^10^)**	**Parameters (10^6^)**
CycleGAN (Zhu et al., 2017a)	22.76	45.52
MUNIT (Huang et al., 2018)	30.95	60.12
WarpGAN (Shi et al., 2019)	**3.38**	**22.68**
CariMe (Gu et al., 2021)	15.20	56.44
Ours	3.63	26.2

Best and Second Best Results are highlighted and underlined.

## 5. Conclusion

In this paper, we propose a Style Attention based Global-local Aware GAN for Personalized Facial Caricature Generation, which applies the characteristics of a subject to generate personalized caricature. To integrate the facial characteristics of a subject, we introduce a landmark-based warp controller for personalized shape exaggeration, which employs the facial landmarks as control points to warp image according to its facial features. To fuse the facial feature with caricature style appropriately, we introduce a style-attention module, which adopts an attention mechanism, instead of the simple Adaptive Instance Normalization, to perform style transfer. To reduce the bad cases and increase the quality of generated caricatures, we propose a multi-scale discriminator to both globally and locally discriminate the synthesized and real caricature, which improves the whole structure and realistic details of the synthesized caricature. Furthermore, we qualitatively and quantitatively evaluate our proposed method on both WebCaricature and CaVINet dataset and empirically prove that our proposed method achieves the best performance among the compared methods. Since the multi-scale discriminator classifies the identities of the photo, the generated caricature and the real caricature, our method requires the caricature dataset with multiple photo-caricature pairs from the same identity. In future work, we will redesign the identity classification part. Specifically, we will extract the identity features of the given images and adopt a classifier to classify the identity without considering whether it is photo, the generated caricature or the real caricature.

## Data availability statement

Publicly available datasets were analyzed in this study. This data can be found here: https://cs.nju.edu.cn/rl/WebCaricature.htm; https://github.com/lsaiml/CaVINet.

## Author contributions

XZ was responsible to study conception and design. WC took in charge of the data collection. WX was in charge of the analysis and interpretation of results. LS drafted the manuscript. All authors reviewed the results and approved the final version of the manuscript.
